# Target Cueing Provides Support for Target- and Resource-Based Models of the Attentional Blink

**DOI:** 10.1371/journal.pone.0037596

**Published:** 2012-05-22

**Authors:** Hannah L. Pincham, Dénes Szűcs

**Affiliations:** Department of Experimental Psychology, University of Cambridge, Cambridge, United Kingdom; University of Groningen, The Netherlands

## Abstract

The attentional blink (AB) describes a time-based deficit in processing the second of two masked targets. The AB is attenuated if successive targets appear between the first and final target, or if a cueing target is positioned before the final target. Using various speeds of stimulus presentation, the current study employed successive targets and cueing targets to confirm and extend an understanding of target-target cueing in the AB. In Experiment 1, three targets were presented sequentially at rates of 30 msec/item or 90 msec/item. Successive targets presented at 90 msec improved performance compared with non-successive targets. However, accuracy was equivalently high for successive and non-successive targets presented at 30 msec/item, suggesting that–regardless of whether they occurred consecutively–those items fell within the temporally defined attentional window initiated by the first target. Using four different presentation speeds, Experiment 2 confirmed the time-based definition of the AB and the success of target-cueing at 30 msec/item. This experiment additionally revealed that cueing was most effective when resources were not devoted to the cue, thereby implicating capacity limitations in the AB. Across both experiments, a novel order-error measure suggested that errors tend to decrease with an increasing duration between the targets, but also revealed that certain stimulus conditions result in stable order accuracy. Overall, the results are best encapsulated by target-based and resource-sharing theories of the AB, which collectively value the contributions of capacity limitations and optimizing transient attention in time.

## Introduction

**Figure 1:**
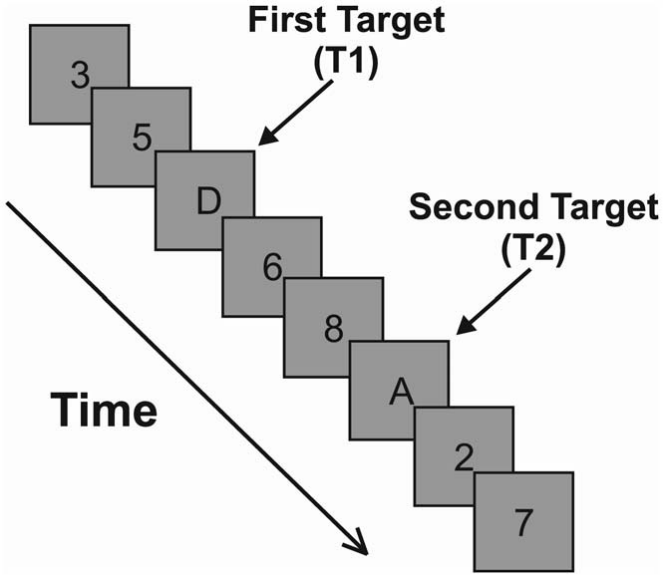
A typical AB paradigm with target letters and digit distractors. Stimuli replace one another in the centre of the monitor at a rate of 100 msec/item. In this figure, T2 accuracy would typically be low because T2 appears at lag 3 (3 items or 300 msec after T1). T1 accuracy is typically at or close to ceiling, regardless of lag.

The attentional blink (AB) describes a deficit in processing the second of two masked targets (T1 and T2) in a rapid serial visual presentation (RSVP) stream [1], [2]. In a typical AB task, target and distractor stimuli replace one another in the centre of a computer screen at a rate of 100 msec/stimulus (see Figure 1). T2 report is typically conditionalised on correct T1 report [2]. T2 detection is impaired if T2 appears 200–600 msec after T1 but T2 is spared from the deficit if it is presented immediately after T1 at lag 1, a phenomenon termed ‘lag1 sparing’ [3], [4]. Although T2 accuracy is reduced if T2 appears within the blink period, the AB is not an exhaustive deficit because T2 remains accurately detected on some trials. Recent work has strengthened this notion by demonstrating that the AB can be easily overcome if a cue is inserted before T2 in the RSVP stream [5], [6], [7], [8]. Here we report an investigation of target-target cueing within the AB. This study aimed to confirm established effects regarding the temporal definition of the AB, and to validate existing cueing phenomena using more rapid stimulus presentation streams than have been previously reported. The current experiments enhanced an understanding of these issues through the employment of novel data analysis techniques and a systematic manipulation of experimental parameters. To achieve our aims, we examined the successive target advantage phenomenon using 30 msec and 90 msec presentation speeds in Experiment 1. In Experiment 2, we examined target-target cueing across four different presentation speeds.

Recent research has demonstrated that the AB can be avoided. A cue placed before T2 dramatically enhances T2 accuracy, even if T2 occurs within the blink period [5], [6], [7], [8]. In this context the ‘cue’ assumes a broad definition and can refer to a target, a stimulus designed to capture attention or another priming event. In order for a cue to increase T2 accuracy, it must share features with T2 or with the participants’ attentional set [6]. For example, a green stimulus will successfully cue a red T2 if participants are required to detect red or green targets. However, the same green stimulus will be an ineffective cue if participants are instructed to attend to red targets only [6]. Interestingly, the cue need not be consciously detected (see [9] for a demonstration of this effect in a slightly different paradigm). An additional target placed before T2 can act as a cue. Therefore, although cueing effects likely contribute to lag 1 sparing (because T1 acts as a cue for T2 [5], [8]), recent reports suggest that cueing may not be the only mechanism underlying high T2|T1 performance at lag 1 (see [10]).

Outside of lag 1 sparing, the most well documented instance of target-target cueing within the AB is the ‘successive target advantage’ [8], [11], [12], [13]. At a 10 Hz presentation rate, the third of three successive targets (TTT) is more accurately detected than is the second of two targets separated by a distractor (TdT). Evidence for this so called ‘extended sparing’ initially presented a challenge to traditional capacity limitation theories of the AB, which argue that the AB is caused by cognitive resources being unduly occupied by T1 (for example, [14]). Extended sparing appears to undermine capacity limitation accounts because resource intensive trials (three-target trials) result in better performance than seemingly less intensive two-target trials. Di Lollo and colleagues developed the Temporary Loss of Control (TLC) model to explain this successive target advantage [5], [11]. TLC is a distractor-based account that suggests the AB arises from an inability to inhibit intervening distractor stimuli. The TLC model argues that T1 encoding causes the participant to lose control over a stimulus filter endogenously set to identify targets. If a distractor is encountered immediately after control is lost, the filter is exogenously re-configured to identify distractors. Consequently, T2 will not match the new filter specifications and may be ‘blinked’ (that is, T2 is lost to conscious awareness and cannot be successfully reported). If successive targets are presented, the input filter is not reset to prioritise distractors, thereby avoiding the blink.

The TLC model is inconsistent with recent findings. For example, Bowman and Wyble [15] examined the AB using stimulus onset asynchronies (SOAs) of 50 msec and 100 msec. Confirming the temporal-based definition, the AB deficit was apparent when T2 appeared 200 msec after T1. This corresponded to lag 2 for the 100 msec SOA condition, and lag 4 for the 50 msec SOA condition. Additionally, the detection of T2 was spared at lag 1 for the 100 msec condition (‘lag 1 sparing’) but at lag 2 for the 50 msec condition. The TLC model has difficulty explaining lag 2 sparing at 50 msec/item. According to TLC, the participants’ input filter would have been reset by the distractor intervening between the two targets (TdT), causing T2 to be blinked.

Bowman, Wyble, Nieuwenstein and colleagues [15], [16], [17], [18] employ the eSTST model to explain lag 2 sparing at 50 msec/item. eSTST is a computational target-based model that builds upon Chun and Potter’s [14] two-stage account of the AB. The two-stage account argues that all stimuli undergo low level visual processing in an early capacity-free stage. The second processing stage is resource limited and encompasses more elaborate mechanisms such as consolidation in working memory. According to the two-stage account, T2 is unable to access the second processing stage because that stage is occupied by T1. As a result, T2 is subject to decay and interference, and may be blinked. The eSTST model specifically argues that a 150 msec blast of transient attention is elicited in response to T1 detection. The transient attentional response fires for a fixed temporal period and enhances the representation of targets falling within that period, regardless of whether distractors also fall in that interval [6]. In this sense, T2 detection can be spared at any lag, provided that T2 occurs during the temporal window of attentional enhancement following T1 [9]. Transient windows of attention can also account for the successive target advantage: each target initiates a transient attentional response such that successive targets effectively generate a sustained state of attention. By contrast, if T2 appears after the blast of attention, it may be unable to access (or is at least impeded in accessing) working memory, and may be blinked. One possibility for this access impairment is because the working memory system, which is busy encoding T1, actively suppresses transient attentional responses to subsequent targets in an attempt to preserve the episodic structure. Alternatively, the attentional boost may possess a grow-and-shrink envelope, which, across time, enhances or dampens target representations.

Distractor-based models of the AB, such as the Boost and Bounce model can also successfully explain the successive target advantage [19]. According to this model, the working memory system makes use of a filter that ‘boosts’ task-relevant information and inhibits distractors in order to prevent them from accessing working memory. The filter attentionally enhances T1 in order for T1 to be consolidated in working memory. However, the T1+1 distractor item is similarly enhanced (because it appears soon after T1). The boosted T1+1 item causes a refractory ‘bounce’ in attention to overcome the fact that a distractor stimulus has been enhanced. It is this bounce that reduces the attention available for T2 processing, hence resulting in the AB. The boost is temporally defined so that representations of items appearing within the boost will be enhanced. The absence of an intervening distractor on successive target trials prevents the occurrence of the inhibitory bounce.

Evidence for the successive target advantage, or extended sparing, is robust [11], [12], [20]. Drawing on the transient attention literature, we argue that the successive target advantage will not emerge when extremely fast presentation rates are used. That is, accuracy for the final target should be equivalent across successive (TTT) and non-successive (TdT) trials with very brief SOAs. For example, using a presentation rate of 30 msec/item, the final target will fall within the window of attention initiated by T1, regardless of whether targets are successive. Equivalent performance across 30 msec successive and non-successive target trials would be consistent with the eSTST model because the transient attentional window (approximately 150 msec duration) will last for longer than the time taken to display three consecutive 30 msec stimuli. By contrast, distractor based models such as TLC would predict poorer performance on non-successive target trials due to the presence of intervening distractor stimuli. Wyble et al. [21] recently presented data relating to this issue. In Experiment 1 of their study, four targets were either successive or alternated with distractor stimuli (TTTT or TdTdTdTd), and presented at 53 msec/item or 107 msec/item. In-keeping with eSTST, the accuracy difference between successive and separated targets was much more pronounced at 107 msec/item. However, the efficacy of the successive target advantage has not been tested using stimulus presentation rates as rapid as 30 msec/item. An examination of such rapid streams is useful to provide convergent validity for extant findings. Additionally, 30 msec/item streams also enable systematic comparisons across presentation rates. In Experiment 2, we used four different RSVP rates, all of which were capable of eliciting the AB deficit, and all of which were multiples of 30 msec.

Results from cueing studies can usefully inform the debate surrounding resource-sharing (or capacity limitation) hypotheses of the AB [20], [22]. The successive target advantage originally undermined resource-sharing accounts because the introduction of a resource consuming target improved final target performance. By contrast, other behavioural and neuroimaging studies clearly support resource sharing accounts, suggesting that the AB is caused by a disproportionate investment of resources to T1 at the expense of T2 [22]. For example, the neural index of resource allocation (the P300 event-related brain potential) is enhanced for T1 and reduced for T2 on trials where T2 is blinked [23], [24]. Taking a slightly different view, we do not see the advantage conferred by target-target cueing as being wholly incompatible with resource sharing accounts of the AB. For example, even though cued T2 trials should result in better performance than uncued T2 trials, T2 performance might be even further enhanced when resources are not devoted to processing the cue itself. In other words, if cueing and resource sharing accounts are compatible, cueing should always benefit T2 processing but T2 accuracy should be *even better* on trials where the cue is not detected. Given the debate surrounding the compatibility of cueing and resource sharing, we investigated this relationship in the current study. It was hypothesised that cueing would enhance T2 accuracy compared with uncued T2 trials. Moreover, T2 performance should be further improved for trials on which the cue was undetected versus trials on which the cue was detected. Such a finding would provide support for resource sharing accounts of the AB.

In addition to measuring target detection accuracy, we calculated the degree of target order-errors using a novel ratio metric that was developed for this purpose. The rationale behind this measure was to provide an additional dependent variable, which is crucial within AB research where accuracy is typically the only available measure. Previous work has shown that the ability to correctly disambiguate target order increases with increasing lag between T1 and T2 [25], [26]. This finding has been recently qualified by Spalek et al. [27] who confirmed that the target order-errors are enhanced during the AB period, even when the temporal distinctiveness between successive targets is held constant. Contemporary investigations into the AB have started to focus efforts on distinguishing between two alternate explanations of order-errors. The episodic-integration explanation posits that targets presented in close succession are processed as a single event [15], [25], [28]. Consequently, temporal information is lost, resulting in order-errors. The prior-entry explanation argues that order-errors are attentionally based, so that attended targets will achieve consciousness earlier than unattended ones [27], [29], [30]. Although the current study was not explicitly designed to distinguish between these two explanations, it examined the degree of order-errors across various lags and presentation speeds in order to provide a systematic examination of order-errors within RSVPs. The employed analyses focused on comparing order-errors inside versus outside the blink period. Further, by subjecting the accuracy and order-errors data to the same statistical analysis, we were able to examine whether these two dependent measures would generate compatible or conflicting results. Such findings should provide insight into whether target identity information (accuracy) and target episodic information (order-errors) are differentially affected during the AB. Given predictions from models such as the eSTST, which is predicated on maximising episodic information at the expense of target accuracy, we expected the AB deficit to be confined to accuracy measures rather than order-error measures.

To summarise, the current study employed target-target cueing and various speeds of presentation to confirm and extend the temporal definition of the AB, and to investigate the relationship between cueing and resource sharing accounts of the AB. Experiment 1 contrasted the predictions of target-based and distractor-based theories of the AB by investigating the successive target advantage across 30 msec and 90 msec presentation rates. At 90 msec presentation rates, the final target in successive trials (TTT) should be better detected than that in non-successive trials (TdT) for the reasons described above. However, at 30 msec presentation rates, we hypothesised that successive and non-successive trials would produce equivalent levels of accuracy because the final target falls within T1’s transient attentional window. Experiment 2 ensured the efficiency of cueing with a 30 msec cue lead time. In Experiment 2, a cueing target was positioned before the final target in order to directly test the facilitatory effect of target-target cueing at 30 msec, 60 msec, 90 msec and 120 msec presentation speeds. The use of various presentation speeds across Experiments 1 and 2 allowed us to validate the temporal based definition of the AB, confirming that the AB is based in time and not in lag [15], [31], [32], [33]. This design also enabled a systematic investigation of target report order-errors across various presentation rates.

## Experiment 1

Experiment 1 investigated the efficacy of the successive target advantage across 30 msec and 90 msec presentation rates. Some investigations into the successive target advantage may have unfairly loaded working memory across conditions because successive target trials contained three targets (TTT) whereas non-successive target trials only contained two targets (TdT). To overcome this potential difficulty, we positioned a third target directly after T2 on half of the experimental trials (see [34]). Importantly, the term “lag” continues to describe the position of T2 relative to T1. The comparison of interest was therefore between T3 accuracy on lag 1 trials (TTT) and T2 accuracy on lag 2 trials (TdTT). The standard two target trials (TdT) were also included for comparison. We hypothesised that successive targets would enhance performance for the 90 msec SOA condition. However, we expected equivalent accuracy for successive and non-successive targets presented at 30 msec because the targets already fall within T1’s window of attention.

Experiment 1 also confirmed the time-course of the AB by de-confounding lag and SOA. Across both SOAs, T2 could occur at lag 1, 2 or 6 (see Table 1). For the 90 msec SOA condition, we predicted that T2 would be spared at lag 1 (90 msec after T1), blinked at lag 2 (180 msec after T1) and should have recovered by lag 6 (540 msec after T1). For the 30 msec SOA condition, T2 should be spared at lags 1 and 2 (30 msec and 60 msec after T1 respectively), and blinked at lag 6 (180 msec after T1). Support for these hypotheses would verify the time-based nature of the AB.

**Table 1:** Example stimuli employed in Experiment 1.

	Three Target Trials	Two Target Trials
Lag 1	**2 5 4 B X R 7 3 2 8 5 4 6 8 2**	**2 5 4 3 8 B X 8 7 2 8 5 4 6 8**
Lag 2	**4 7 6 8 C 3 A N 5 6 9 8 2 6 3**	**4 7 6 8 C 3 A 9 5 6 9 8 2 6 3**
Lag 6	**6 5 7 8 9 V 2 4 9 4 6 E K 3 2**	**6 5 7 V 2 4 9 4 6 E 4 3 2 5 7**

Participants were required to detect target letters within digit distractors. SOA was either 30 msec or 90 msec. The location of T1 was jittered between serial positions 4, 5 and 6. T2 appeared at lag 1, 2 or 6. Every trial contained at least two targets. If a third target appeared, it was positioned directly after T2. Targets are underlined in this table for ease of detection. Targets were not underlined in the actual task.

### Method

#### Participants

Fourteen graduate students from the University of Cambridge participated voluntarily. This study was approved by the Ethical Research Committee at the University of Cambridge, and participants provided written, informed consent. All participants reported normal or corrected-to-normal vision. The participants (6 males) were 25.5 years old on average (SD = 1.74).

#### Stimuli and Apparatus

The experiment was presented on a Sony GDM CRT monitor, refreshing at 100 Hz. Alphanumeric stimuli were generated using Presentation (Neurobehavioural Systems). Targets were letters excluding I, M, O, Q and W. Distractors were single digits excluding 0 and 1. Alphanumeric stimuli were always presented in black, on a white screen. Each alphanumeric stimulus was shown in ‘Arial Rounded Bold’ font, and subtended a visual angle of 3.8° vertically and 2.9° horizontally, assuming a viewing distance of 57 cm.

#### Design and Procedure

On each trial, a fixation cross (subtending 2°×2°) was presented in the centre of the monitor for 500 msec. An RSVP stream of 15 alphanumeric items was then shown in the centre of the monitor, with each RSVP item replacing the preceding one. Trials contained two or three letter targets presented among digit distractors. After each RSVP stream was presented, participants reported the target letters in order of appearance. Participants were given unlimited time in which to make their response, and were required to guess if they were unsure. The identities of the letter targets and the digit distractors were randomly assigned on each trial, with the restriction that successive items were not the same. For this and the subsequent experiment, a target response was deemed correct if the target identity was correctly reported, regardless of order of report.

The experiment contained 4 blocks of 75 trials, totalling 300 experimental trials. SOA (30 msec or 90 msec) was manipulated across blocks. To control for stimulus exposure duration, alphanumeric stimuli were displayed for 30 msec and the interstimulus interval (ISI) varied. The ISI was set at 0 msec for the 30 msec SOA blocks and 60 msec for the 90 msec SOA blocks. During the 60 msec ISI period, no alphanumeric stimulus was presented on the screen.

Numbers of targets per trial were manipulated across blocks. Trials in a given block either contained two or three targets. In order to prevent the predictable occurrence of T1, T1 randomly appeared in serial positions 4, 5 or 6. T2 appeared at lag 1, lag 2 or lag 6. If a third target was present, it occurred directly after T2. Each block contained only one SOA/number of targets combination. The four blocks were therefore: 30 msec/2 targets 30 msec/3 targets, 90 msec/2 targets, 90 msec/3 targets. The order in which participants received these four blocks was counterbalanced. Additionally, the order of the trials within each block was randomised.

Participants were explicitly told whether a given block would contain two or three target trials. Each block was preceded by ten practice trials, during which time the experimenter was present. Testing occurred individually in a sound-attenuating booth. Example RSVP streams are shown in Table 1.

#### Data Analysis

To examine the successive target advantage (Analysis 1), we employed a repeated measures ANOVA with SOA (90 msec, 30 msec), target position (serial position 1 (TTT or TdTT), serial position 3 (TTT or TdTT) and trial type (successive, non-successive) as factors. To examine the time-based nature of the AB (Analysis 2), T1 and T2 accuracy scores were separately subjected to a repeated measures ANOVA with SOA (30 msec, 90 msec) and lag (1, 2, 6) as factors. For all ANOVAs, Tukey post hoc contrasts were used to probe significant interaction effects and effect sizes were approximated using *η^2^*.

Target order report was considered using a novel order-error ratio variable. This measure provides an indication of the degree to which order-errors have been made in a given condition. The measure was defined for n-target trials using the following formula:
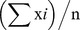
where n = number of targets, x_i_ = 1 if the participant correctly detected the i^th^ target but reported it in the incorrect position, and x_i_ = 0 otherwise. x_i_ assumed a value of 0 for correctly identified targets in their correct position, and for incorrectly identified targets. The order-error ratio could therefore range from 0–1, where 0 represents no order-errors and 1 represents maximum order-errors. A value of 0 indicates that all correctly-identified targets were reported in their correct location and a value of 1 reflects all correctly-identified targets being reported in an incorrect location. In this manner, the order-error ratio applies to trials with partially correct target identity reports (for example, T1 correctly identified and T2 incorrect identified, or T1 and T3 correctly identified and T2 incorrectly identified) as well as trials where all targets were correctly identified. The ratio is valid for all n-target trials and can therefore be used to compare target order order-errors across trials with unequal target numbers.

Order-errors were analysed using a repeated measures ANOVA with SOA (30 msec, 90 msec) and lag (1, 2, 6) as factors (Analysis 3). Because the order-error ratio was designed to allow comparison across trials with various numbers of targets, data from both two-target and three-target trials were included.

### Results

**Figure 2:**
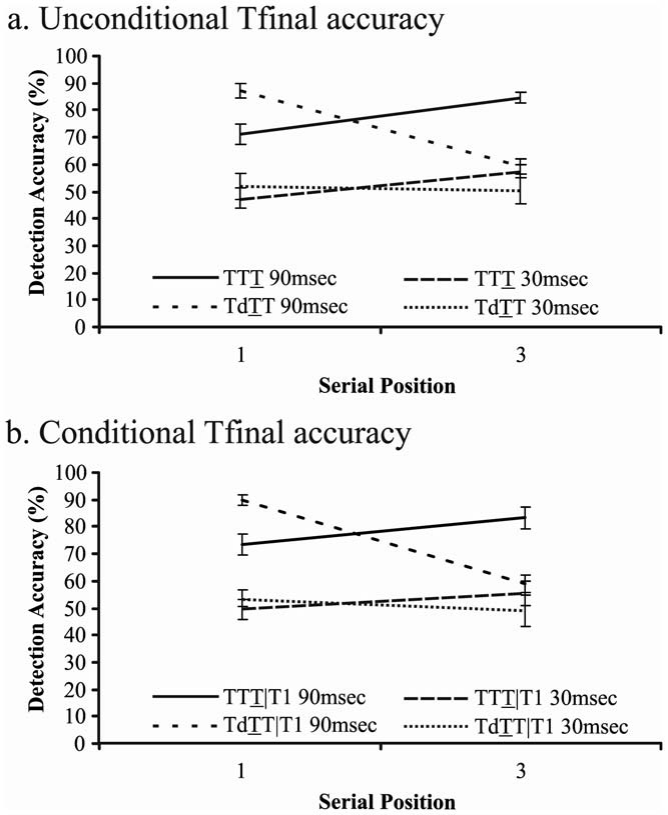
Target detection accuracy for targets in serial position 1 (TTT or TdTT) and serial position 3 (TTT or TdTT). Data from the 30 msec SOA and 90 msec SOA conditions are shown. (a) represents unconditional Tfinal accuracy. (b) represents Tfinal accuracy conditionalised on T1 detection. Error bars represent standard errors.

Analysis 1: As shown in Figure 2a, unconditional accuracy scores from three-target trials (TTT and TdTT) were entered into the SOA × target position × trial type ANOVA described above. This analysis yielded a main effect of SOA, indicating that accuracy was higher on 90 msec SOA trials (SOA: F(1,13) = 86.502, p<.001, *η^2^* = .869). The SOA × target position and target position × trial type interactions were also significant (F(1,13) = 6.183, p = .027, *η^2^* = .322; F(1,13) = 34.904, p<.001, *η^2^* = .729). Importantly, the three-way interaction effect was highly significant (F(1,13) = 15.606, p = .002, *η^2^* = .546), indicating that the difference between successive and non-successive trials was larger for 90 msec trials than for 30 msec trials. Tukey post-hoc comparisons between successive and non-successive trials were employed to probe the three-way interaction. On 90 msec SOA trials, successive targets improved detection accuracy for targets presented at serial position 3 (p<.001). However, successive targets hindered T1 accuracy (p = .019). In other words, the original successive target advantage [11] was replicated. By contrast, in the 30 msec SOA condition, target detection accuracy was equivalent across successive and non-successive trials at serial position 1 (p = .960) and at serial position 3 (p = .647). Even when more lenient t-test comparisons were employed on the 30 msec data, there remained no difference between successive and non-successive trials at serial position 1 (p = .271) or at serial position 3 (p = .154). Consequently, there was no evidence of a successive target advantage when items were presented at 30 msec/item. Notably, the same pattern of results was obtained regardless of whether the non-successive trials contained two (TdT) or three (TdTT) targets (three-way interaction using two-target trials: F(1,13) = 19.351, p = .001, *η^2^* = .598). Further, as shown in Figure 2b, the results were unchanged when target accuracy was conditionalised on T1, that is, TTT|T1 and TdTT|T1 (three-way interaction: F(1,13) = 9.932, p = .008, *η^2^* = .433).

**Figure 3:**
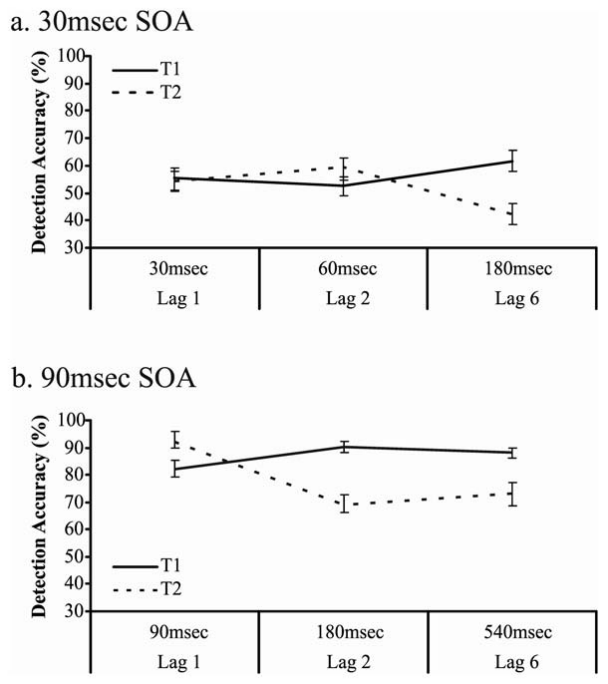
Target detection accuracy for T1 and T2 across the three lag conditions. (a) displays the 30 msec SOA trials. (b) displays the 90 msec SOA trials. The time points displayed on the x-axis are times between T1 onset and T2 onset. Error bars show standard errors of the mean.

Analysis 2: For convenience, we used unconditional data from the two target trials to confirm the time-based definition of the AB. However, the exact same pattern of ANOVA results was produced regardless of whether two-target or three-target trials were used and regardless of whether the data was unconditional or conditionalised on T1 (T2|T1). As shown in Figure 3, the SOA × lag ANOVA conducted on the T2 accuracy scores revealed main effects of SOA and lag, and a significant interaction effect (SOA: F(1,13) = 78.886, p<.001, *η^2^* = .859; lag: F(2,26) = 13.438, p<.001, *η^2^* = .508; interaction: F(2,26) = 28.272, p<.001, *η^2^* = .685). Tukey post hoc comparisons revealed that, for the 90 msec SOA condition, T2 accuracy was higher at lag 1 than lag 2 and 6 (p<.001) and did not differ between lags 2 and 6 (p = .782). For the 30 msec condition, T2 accuracy was equivalent across lags 1 and 2 (p = .722), and was significantly reduced at lag 6 compared with lag 2 (p<.001).

T1 accuracy was analysed to examine whether changes in T2 accuracy are accompanied by changes in T1 accuracy (see Figure 3). The SOA × lag ANOVA yielded main effects of SOA and lag, as well as a significant interaction effect (SOA: F(1,13) = 265.316, p<.001, *η^2^* = .953; lag: F(2,26) = 16.317, p<.001, *η^2^* = .557; interaction: F(2,26) = 3.750, p = .037, *η^2^* = .224). Tukey post hoc comparisons confirmed that, for the 90 msec SOA condition, T1 accuracy was reduced on lag 1 trials compared with lag 2 (p<.001), but did not differ between lag 2 and lag 6 trials (p = .790). For the 30 msec condition, T1 accuracy was equivalent across lags 1 and 2 (p = .789), and was significantly improved at lag 6 compared with lag 2 (p = .019).

**Figure 4:**
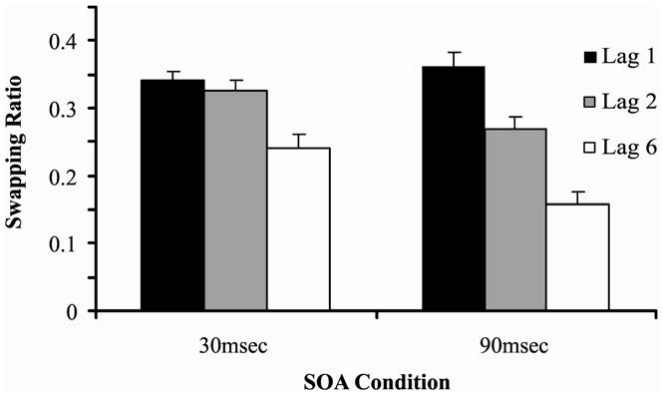
Order-error ratios across lags 1, 2 and 6 for the 30 msec SOA and 90 msec SOA conditions. The order-error ratio ranges from 0 (no order-errors) to 1 (all correctly identified targets order-error). Across both SOAs, order-errors were least frequent at lag 6. Error bars represent standard errors.

Analysis 3: Target-report order-errors were analysed using a lag × SOA ANOVA. As shown in Figure 4, order-errors were more frequent for the 30 msec SOA trials (F(1,14) = 11.667, p = .005, *η^2^* = .473). The lag main effect also yielded significant results (F(2,26) = 77.386, p<.001, *η^2^* = .856). However, these main effects were qualified by an interaction effect (F(2,26) = 5.552, p = .010, *η^2^* = .299). Tukey pairwise contrasts revealed that, for the 90 msec SOA trials, the degree of order-errors was higher on lag 1 trials compared with lag 2 and lag 6 trials (p = .005 and p<.001 respectively). Order-errors were also higher on lag 2 trials compared with lag 6 trials (p<.001). For the 30 msec SOA condition, the degree of order-errors was equivalent between lag 1 and lag 2 trials (p = .974) but was higher on lag 1 and lag 2 trials compared with lag 6 trials (p = .002 and p = .011 respectively).

### Discussion

The successive target analysis produced clear findings. At the 90 msec presentation rate, successive targets improved accuracy for targets in serial position 3 (TTT>TdTT). By contrast, successive targets diminished T1 accuracy (TTT<TdTT). These results held regardless of whether a third target followed T2 on non-successive trials (TdT versus TdTT). We were therefore able to replicate Di Lollo et al. [11] and confirm that the successive target advantage is valid when numbers of targets are equated across successive and non-successive trials. Clearly, working memory differences due to unequal numbers of targets across trials were not responsible for Di Lollo et al.’s original findings.

The significant interaction between the 30 msec and 90 msec presentation rates revealed that the AB deficit was not present for time ranges very close to T1. In other words, successive targets did not provide a substantive benefit if the successive targets appeared within 100 msec of T1 (as in the 30 msec condition). Although these data are consistent with recent findings reported by Wyble et al. [21], a distinction can be made between the two studies. The fastest presentation speed employed by Wyble et al. was more than 1.5 times slower than the 30 msec/item speed employed here. As a result of the 30 msec/item RSVP streams, three targets could be successively presented within the same time-frame typically required for a single item to be displayed. We were therefore able to demonstrate ‘lag 2 sparing’ and were confident that this effect was operating over the same temporal parameters as T1 detection in a standard AB paradigm. Clearly, this particular finding is only possible using 30 msec/item (or faster) stimulus streams.

This data obtained in Experiment 1 conforms to our hypothesis and may be taken as evidence that at the 30 msec presentation rate, T2 and T3 fell within the window of attentional enhancement initiated by T1. However, a potential caveat to the above explanation is that the 30 msec successive targets may not have provided enough lead time to achieve an accuracy improvement. In other words, there is a chance that the 30 msec cues may simply be ineffective. Indeed, transient attention research indicates that the optimal cue-target SOA exceeds 30 msec [35], [36], [37]. This possibility was investigated in Experiment 2.

Experiment 1 also supports and extends the notion that the AB deficit is governed by time. Results from the 90 msec SOA condition largely mirrored traditional AB results – T2 was spared at lag 1 and suffered at lag 2 [2], [14]. Notably, T2 accuracy had not recovered by lag 6. This probably occurred because T2 was positioned too close to T1 in Experiment 1 (540 msec after T1). T2 was positioned at lag 8 in Experiment 2 and this resulted in the typical recovery effect. Importantly, the 30 msec SOA condition in Experiment 1 confirmed that the AB is time based. At the 30 msec presentation rate, T2 was spared at lags 1 and 2 and accuracy was reduced at lag 6.

For both SOA conditions, the T1 accuracy data complemented the T2 findings, indicating that an increase in T2 accuracy was accompanied by a decrease in T1 accuracy. These data may therefore be taken to support resource-sharing accounts of the AB [22], [23], [38], [39].

As expected, the order-errors data did not follow the same pattern as target detection accuracy. If episodic target information were subject to the AB in the same manner as target accuracy, we would predict poorer performance (more order-errors) during the AB. Instead, order-errors tended to decline as the duration between T1 and T2 increased. Interestingly, order-errors tended to decrease with increasing lag [25], [26]. However, the degree of errors was statistically equivalent at lag 1/30 msec and lag 2/30 msec – trials where T2 appeared before the onset of the AB deficit. The contribution of order-errors data to the AB is further examined in Experiment 2.

## Experiment 2

Experiment 2 was designed to investigate why, in Experiment 1, the 30 msec successive cueing manipulation was unsuccessful. The manipulation may have been unsuccessful because, as hypothesised, the 30 msec successive targets fell within the window of attention generated by T1 and performance was already at ceiling. Alternatively, the manipulation may have been unsuccessful because 30 msec is a suboptimal cue lead time [36]. By using a 30 msec SOA condition, Experiment 2 was able to examine whether an immediately preceding 30 msec cue is *ever* capable of enhancing target detection within the AB paradigm. If 30 msec cues *are* able to improve performance, the absence of a 30 msec successive target advantage in Experiment 1 must result from the fact that T2 and T3 fell within T1’s attentional window, and not because 30 msec cues are sub-optimal.

Experiment 2 was also designed to systematically uncover the relationship between cueing efficacy, SOA and lag in an AB task. To that end, we directly examined cueing at four presentation rates: 30 msec, 60 msec, 90 msec and 120 msec. Using a simple cueing paradigm, an additional cueing target (Tcue) appeared before the final target (Tfinal) on cueing trials. Accuracy for Tfinal was compared with accuracy for an uncued Tfinal, which was not preceded by an additional target. Consequently, some trials contained three targets (T1, Tcue, Tfinal) and other trials contained only two targets (T1 and Tfinal).

Experiment 2 provided another opportunity to confirm that the AB is a time-based deficit. By manipulating SOA and using two Tfinal lags (lags 3 and 8), Experiment 2 was able to sample a number of temporal intervals. We predicted that, regardless of the speed of presentation, the AB deficit would be governed by time and not lag.

### Method

#### Participants

Nineteen graduate students from the University of Cambridge were compensated £7 for their participation. This study was approved by the Ethical Research Committee at the University of Cambridge, and participants provided written, informed consent. All participants reported normal or corrected-to-normal vision. One participant was excluded for failing to reach a 25% accuracy criterion. The remaining participants (8 males) were 24.2 years old on average (SD = 2.1).

#### Design and Procedure

All experimental details were the same as those in Experiment 1, except as noted. The design employed in Experiment 2 is shown in Table 2. The experiment contained 4 blocks of 100 trials, totalling 400 experimental trials. Across the four blocks, SOA was manipulated as 30 msec, 60 msec, 90 msec or 120 msec. Stimulus exposure duration was held constant at 30 msec with ISI set at 0 msec, 30 msec, 60 msec and 90 msec respectively. Each block contained a single SOA condition. The order of blocks was counterbalanced across participants.

**Table 2:** Example stimuli employed in Experiment 2.

	Cued Trials	Uncued Trials
Lag 3	**2 5 4 B 6 C S 3 2 8 5 4 6 8 2 4**	**2 5 4 3 8 B 7 8 X 2 8 5 4 6 8 9**
Lag 8	**4 7 6 8 C 3 5 6 9 8 2 Y T 6 3 6**	**4 7 6 8 C 3 9 5 6 9 8 2 L 6 3 9**

Participants were required to detect target letters within digit distractors. SOA was either 30 msec, 60 msec, 90 msec or 120 msec. The location of T1 was jittered between serial positions 4, 5 and 6. Tfinal appeared at lag 3 or 8. Every trial contained at least two targets. A third target appeared on cued trials and was positioned directly before Tfinal. The term lag always described the number of positions between T1 and Tfinal. Targets are underlined in this table for ease of detection. Targets were not underlined in the actual task.

Every trial contained 16 RSVP alphanumeric stimuli, two or three of which were target letters. Participants were not told how many targets a given trial would contain, but the program only asked them to provide a third response if three targets had appeared. As in Experiment 1, T1 randomly appeared in serial position 4, 5 or 6. The final target in each trial appeared at lag 3 or lag 8. If a third target occurred, it was shown immediately before Tfinal, as a cue for the final letter (Tcue). Therefore, two-target trials were designated as ‘uncued’ trials whereas three-target trials were ‘cued’ trials. Each block contained an equal number of lag 3/cued, lag 8/cued, lag 3/uncued and lag 8/uncued trial types. These trial types were randomised within each block.

#### Data Analysis

Analysis 1: In order to examine Tfinal accuracy across SOA, a repeated measures ANOVA was employed with SOA (30 msec, 60 msec, 90 msec, 120 msec), lag (lag 3, lag 8) and cueing (cued, uncued) as factors. Tfinal accuracy was conditionalised on correct T1 detection (Tfinal|T1). A follow-up analysis investigated whether cueing efficacy was modulated by detection of the cue. We calculated Tfinal accuracies conditional on T1 and the cue being correctly detected (Tfinal|TĺTcue) or conditional on T1 being detected but the cue being undetected (Tfinal|T1∼Tcue). We then calculated difference scores between each of these values and uncued accuracy (uncued_Tfinal|T1). A positive difference score indicates that cueing *improved* Tfinal accuracy whereas a negative difference score indicates that cueing *impaired* Tfinal accuracy. In this manner, cueing efficacy could be directly contrasted according to whether or not the cue was detected. The data were entered into a three-way ANOVA with difference score (cue detected vs cue undetected), SOA and lag as factors. We were also interested in examining whether cueing caused a deficit in T1 processing. To that end, an ANOVA using T1 accuracy with SOA, lag and cueing as factors was also employed.

Analysis 2: To reinforce the time-based nature of the AB, we examined the relationship between SOA and lag. We used data from two-target trials, which were not contaminated by cueing effects. Unconditional accuracy from each of the four presentation rates were analysed separately, using a repeated measures ANOVA with target (T1, Tfinal) and lag (lag 3, lag 8) as factors. Finally, the target-report order-error ratio introduced in Experiment 1 was used to measure the degree of order-errors in Experiment 2. As in Experiment 1, data from two-target (uncued) and three-target (cued) trials were included. The order-errors data were analysed using a repeated measures ANOVA with SOA and lag as factors (Analysis 3).

### Results

**Figure 5:**
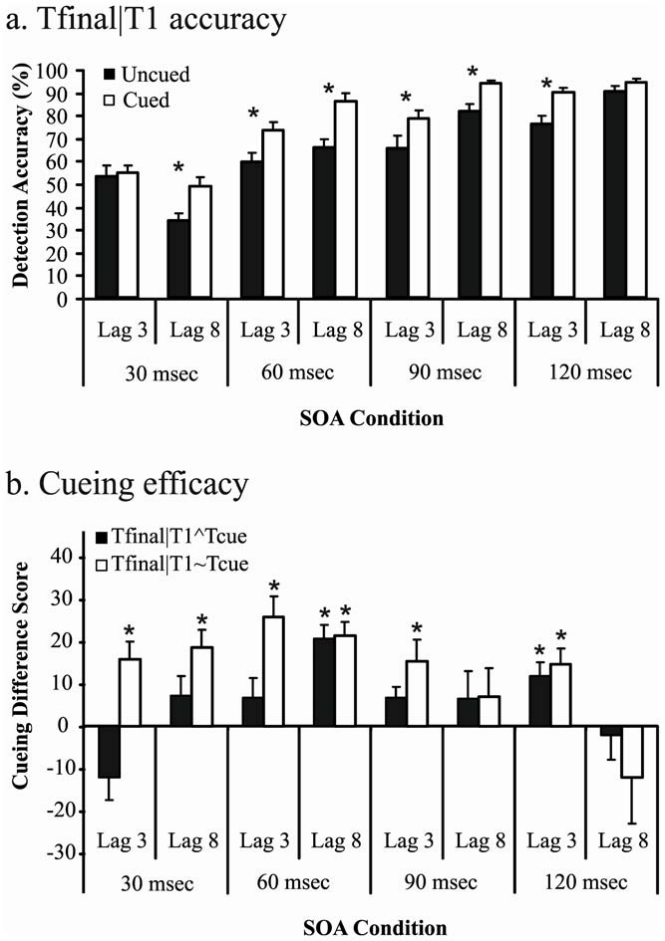
Target detection accuracy for Tfinal across every combination of lag and SOA. (a) displays Tfinal|T1 accuracy across cued and uncued trials. Asterisks indicate a significant difference between cued and uncued trials. Tfinal accuracy was significantly improved on cued trials for all comparisons except 30 msec/lag3 and 120 msec/lag8. (b) displays Tfinal cueing efficacy scores. These scores were conditionalised on T1 and the cue being correctly detected (Tfinal|TĺTcue) or on T1 being identified but Tcue being incorrectly detected (Tfinal|T1∼Tcue). A positive difference score indicates a benefit for cued trials over uncued trials. A negative difference score indicates a benefit for uncued trials over cued trials. Asterisks indicate a significant deviation from 0, where 0 represents equivalent accuracy across cued and uncued trials. Error bars show standard errors of the mean.

Analysis 1: As shown in Figure 5a, Tfinal|T1 accuracy scores were entered into a lag× SOA × cueing ANOVA. This analysis yielded main effects of SOA, lag and cueing (SOA: F(3,51) = 138.502, p<.001, *η^2^* = .891; lag: F(1,17) = 6.455, p = .021, *η^2^* = .275; cueing: F(1,17) = 53.281, p<.001, *η^2^* = .758). A significant interaction between SOA and cueing indicated that the cueing benefit was not equivalent across SOAs (F(3,51) = 3.618, p = .021, *η^2^* = .175). Notably, these effects were qualified by a three-way interaction (F(3,51) = 5.312, p = .003, *η^2^* = .238). Tukey posthoc comparisons indicated that, for the 30 msec SOA condition, cueing enhanced Tfinal accuracy at lag 8 (p = .001) but not lag 3 (p = 1.000). For the 60 msec SOA and the 90 msec SOA conditions, cueing was effective at both lags (60 msec: p = .003, p<.001; 90 msec: p = .001, p = .011). For the 120 msec SOA condition, cueing was only effective at lag 3 (p = .003) but not lag 8 (p = .983). In other words, cueing was always effective, except for the temporally extreme conditions: lag 3 for the 30 msec SOA and lag 8 for the 120 msec SOA. Notably, the exact same pattern of results was obtained if Tfinal accuracy was not conditionalised on T1.

To examine whether detection of the cue impacts Tfinal accuracy, a cue-detection × SOA × lag ANOVA was employed (Figure 5b). This analysis revealed that cueing was stronger when the cue was not detected (F(1,17) = 8.097, p = .011, *η^2^* = .322). The size of cueing also differed according to SOA (F(3,51) = 4.215, p = .010, *η^2^* = .199). The three-way interaction was not significant (F<1). To investigate whether cueing provided a significant benefit to Tfinal accuracy, we employed post-hoc contrasts that tested whether each difference score differed from 0. For Tfinal|TĺTcue trials, cued trials resulted in significantly enhanced Tfinal accuracy compared with uncued trials at 60 msec/lag8 and 120 msec/lag3. For Tfinal|T1∼Tcue trials, cueing always significantly enhanced accuracy compared with uncued trials, except at 90 msec/lag8 and 120 msec/lag8. Under no conditions did uncued trials generate significantly better accuracy than cued trials (that is, no difference scores were significantly less than 0).

**Figure 6:**
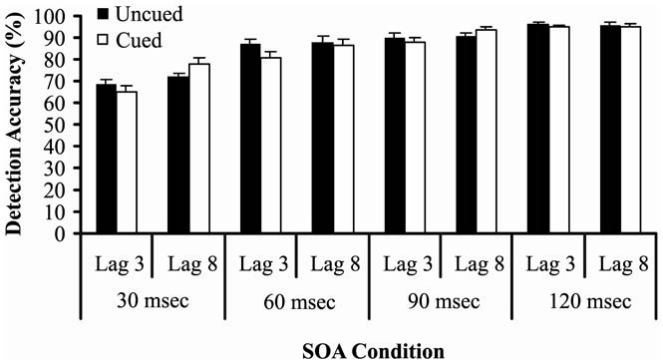
T1 accuracy across every combination of lag and SOA. T1 accuracy did not differ between cued and uncued trials. Error bars show standard errors of the mean.

T1 accuracy scores are shown in Figure 6. The lag × SOA × cueing ANOVA revealed main effects of SOA and lag (SOA: F(3,51) = 78.143, p<.001, *η^2^* = .821; lag: F(1,17) = 22.326, p<.001, *η^2^* = .568). Two interaction effects also yielded significant effects (SOA × lag: F(3,51) = 4.312, p = .009, *η^2^* = .202; cueing × lag: F(1,17) = 5.998, p = .025, *η^2^* = .261). The SOA × lag interaction indicated that, for the 30 msec SOA condition, T1 accuracy was significantly reduced at lag 3 compared with lag 8 (p<.001) but T1 accuracy did not differ across lags for the other three SOA conditions (60 msec: p = .459, 90 msec: p = .417, 120 msec: p = 1.000). The cueing × lag interaction revealed that the effect of cueing on T1 accuracy was stronger at lag 3 than at lag 8. No other effects achieved statistical significance (largest F = 2.109).

**Figure 7:**
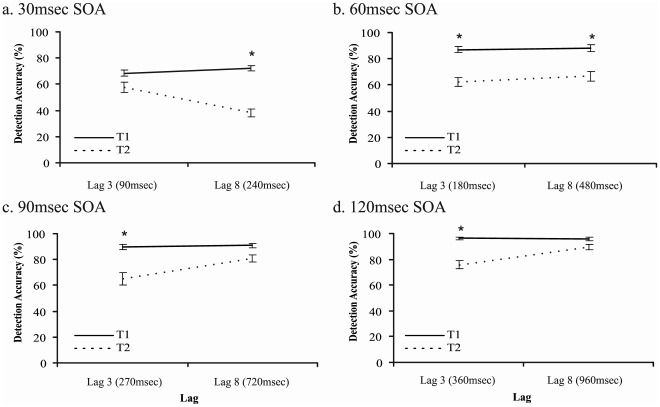
Target detection accuracy for T1 and T2. Accuracies are shown for lags 3 and 8 across four SOA conditions: 30 msec (a), 60 msec (b), 90 msec (c) and 120 msec (d). Asterisks indicate a significant difference between T1 and T2 detection accuracy. Error bars represent standard errors of the mean.

Analysis 2: As shown in Figure 7, a target × lag ANOVA was applied to each SOA condition in order to confirm the time-based definition of the AB. Unconditional accuracy from the two-target trials was used to avoid cueing influences in this temporal analysis. Notably, the exact same pattern of results was obtained if conditional Tfinal accuracy scores (Tfinal|T1) were used instead of unconditional Tfinal accuracy. For every SOA condition, the target main effect was significant because T1 was more accurately detected than Tfinal (p<.001 for every SOA). For the 30 msec SOA condition (Figure 7a), target detection accuracy was higher on lag 3 trials than lag 8 trials (F(1,17) = 14.386, p<.001, *η^2^* = .458). The interaction between SOA and lag was also significant (F(1,17) = 16.994, p<.001, *η^2^* = .500). Tukey post hoc comparisons on the interaction effect confirmed that T1 accuracy did not differ across lags 3 and 8 (p = .802). However, in line with time-based explanations of the AB, Tfinal accuracy was significantly reduced at lag 8 (240 msec after T1) versus lag 3 (90 msec after T1) (p = .001). For the 60 msec SOA condition (Figure 7b), target detection accuracy was equivalent across lags 3 (180 msec after T1) and 8 (480 msec after T1) (F(1,17) = 2.303, p = .147, *η^2^* = .119). Additionally, the interaction between target and lag was not significant (F<1). For both the 90 msec SOA and 120 msec SOA conditions (Figures 7c and 7d), target detection accuracy was significantly better on lag 8 trials than on lag 3 trials (90 msec: F(1,17) = 11.344, p = .004, *η^2^* = .400; 120 msec: F(1,17) = 9.551, p = .007, *η^2^* = .350). The interactions between SOA and lag were also significant (90 msec: F(1,17) = 19.131, p<.001, *η^2^* = .529; 120 msec: F(1,17) = 17.873, p = .001, *η^2^* = .513). Tukey post hoc comparisons on the interaction effect indicated that T1 accuracy did not differ across lags 3 and 8 (90 msec: p = .966, 120 msec: p = .998). But conforming to time-based explanations of the AB, T2 accuracy was significantly improved at lag 8 versus lag 3 (90 msec: p<.001; 120 msec: p<.001). This result suggests that the absence of recovery at lag6/90 msec in Experiment 1 was due to the use of lag 6 rather than lag 8.

**Figure 8:**
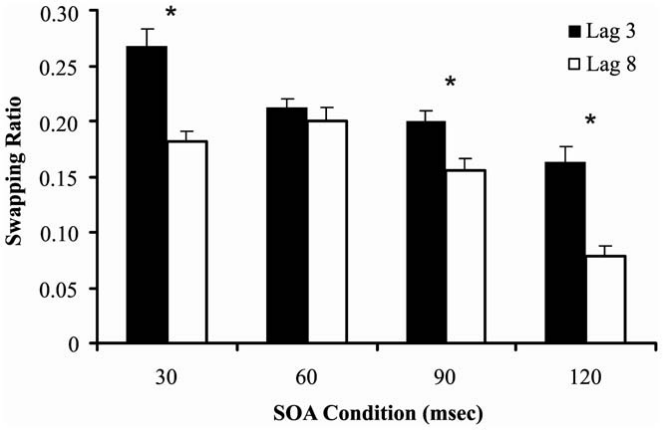
Order-error ratios for lags 3 and 8, across four SOA conditions (30 msec, 60 msec, 90 msec, 120 msec). Asterisks indicate a significant difference between order-errors at lag 3 and lag 8. Error bars represent standard errors of the mean.

Analysis 3: Figure 8 shows the order-errors data employed in the SOA × lag ANOVA. The SOA main effect was significant (F(3,51) = 34.444, p<.001, *η^2^* = .670). The lag main effect was also significant because order-errors was more pronounced at lag 3 than lag 8 (F(1,17) = 127.001, p<.001, *η^2^* = .882). These main effects were qualified by a significant interaction (F(3,51) = 5.856, p = .002, *η^2^* = .256). Tukey post hoc comparisons on the interaction effect indicated that order-errors was significantly increased at lag 3 versus lag 8 for all SOA conditions except 60 msec (30 msec: p<.001, 60 msec: p = .990, 90 msec: p = .018, 120 msec: p<.001). Notably, the same pattern of results was obtained if only the two-target or only the three-target trials were analysed. This provides support for the order-error ratio metric, which is designed to apply across trials and with varying numbers of targets.

### Discussion

Experiment 2 enhances an understanding of cueing within the AB paradigm. For all conditions excluding the earliest and latest conditions (30 msec/lag3 and 120 msec/lag8), Tfinal accuracy was improved if another target letter appeared immediately before Tfinal in the RSVP stream. Experiment 2 therefore allows for a number of important conclusions regarding cueing mechanisms in the AB. First, the AB is not an irreversible deficit but can be easily overcome by inserting another target before the target of interest. Although this general finding has been shown by Kawahara et al. [5] and Olivers et al. [8], it has not been systematically demonstrated across various SOAs. Second, the cue lead time can be as brief as 30 msec duration. Third, the cue can be another target letter (see [9] for similar findings outside the AB paradigm). This is significant because performance was enhanced on cued trials, despite the fact that working memory load may have been higher on cued trials versus uncued trials (detecting three targets versus two targets respectively). Finally, the effect of cueing appears to be automatic because participants were not told about the presence of a cueing target or its potentially beneficial effects.

Experiment 2 validated the findings from Experiment 1. Because Experiment 2 demonstrated that cues with 30 msec lead time were capable of improving performance, the absence of a 30 msec successive target advantage in Experiment 1 was not due to 30 msec cues being sub-optimal. Instead, target detection performance was likely at ceiling in Experiment 1 because the trailing targets fell within the transient window of attention initiated by T1.

Conditional accuracy measures have been shown to be crucial in studies of extended sparing within the AB [40]. In our study, conditional Tfinal data revealed that although cueing was generally effective regardless of whether or not the cue was detected, cueing efficacy was enhanced when the cue was *not* detected. As such, the mere presence of (rather than the detection of) a target stimulus is beneficial [6], [41], [42], [43]. The fact that cueing was stronger when the cue was undetected suggests a possible role for resource sharing in the attentional blink. When the cue consumed resources for detection, the benefit of cueing was reduced. That is, the process of consolidating the cue diverted the attentional resources required for Tfinal detection. As noted above, however, the addition of a cueing target was typically not detrimental. Regardless of whether or not the cue was detected, cued Tfinal accuracy exceeded uncued Tfinal accuracy for all conditions except lag 3/30 msec and lag 8/120 msec (where accuracy was equivalent across cued and uncued trials). Cueing was probably unsuccessful at lag 3/30 msec because Tfinal fell within the window of attention initiated by T1, regardless of whether Tfinal was cued. Cueing was probably unsuccessful at lag 8/120 msec because Tfinal accuracy was already at ceiling.

Similarly to Experiment 1, Experiment 2 provides support for time based models of the AB. Across various SOAs and lags, Tfinal was blinked when it fell 200–500 msec after T1. Interestingly, Tfinal was spared from the AB at lag 3/30 msec. Such ‘lag 3 sparing’ confirms the time based nature (rather than lag based) of the AB. Lag 3 typically represents the peak of the deficit, yet at 30 msec presentation speeds, lag 3 corresponds to 90 msec after T1– a time point before the AB begins.

With regards to target report order-errors, order-errors typically decreased as target onset asynchrony increased. The degree of order-errors was consistent for the 60 msec condition, where both lag 3 and lag 8 fell within the blink period. This finding resonates with Experiment 1, where order-errors were constant across trials where targets occurred before the onset of the blink period (lags 1 and 2 at 30 msec/item). Hence, it may be the case that order-errors tend to decrease as target onset asynchrony increases, yet errors are equivalent when targets are fully within or fully outside the blink period.

## Discussion

Experiments 1 and 2 collectively reveal that the AB is not a ballistic deficit invariably triggered by the occurrence of T1. Rather, the AB can be influenced by target-target cueing. Experiment 1 revealed that successive targets enhance accuracy when stimuli are presented at 90 msec/item but not at 30 msec/item. This absence of a 30 msec/item successive target advantage was validated in Experiment 2. Experiment 2 further demonstrated that cueing is effective both inside and outside the blink period, provided that target detection accuracy is not already at ceiling. In addition, both experiments confirmed the time-based nature of the AB by employing various SOAs and sampling a range of temporal intervals. Collectively, Experiments 1 and 2 help to verify well-established effects in the AB deficit. As discussed below, the current findings are relevant to four issues of theoretical relevance to the AB, including the time-course of the deficit, cueing, resource-sharing and order-errors.

### The Time-course of the AB

This study confirms the time-based nature of the AB using various RSVP speeds. A number of empirical investigations have demonstrated that the AB deficit is time-based by de-confounding SOA and lag [15], [31], [32], [33], [44], [45]. Additional evidence supporting the temporal nature of the AB has been more recently provided by Nieuwenstein and colleagues [17], [46]. In the current study, target detection accuracy was reduced when Tfinal fell within a broadly defined AB period. In Experiment 1, the blink occurred when T2 was positioned 180 msec after T1 (lag6/30 msec and lag2/90 msec) or 540 msec after T1 (lag3/60 msec). In Experiment 2, the blink occurred when T2 was presented anywhere between 180 msec after T1 and 480 msec after T1. By contrast, targets were spared when they appeared before the blink onset. This corresponded to lag 2/30 msec in Experiment 1, and lag 3/30 msec in Experiment 2. Of particular interest, Experiment 2 demonstrated that an uncued T2 survives the blink despite two distractors having intervened between the first and second targets (lag 3/30 msec). In a demonstration of the robust nature of these findings, the results held regardless of whether T2 or Tfinal accuracy was conditionalised on T1. In our opinion, this study is more consistent with target-based, rather than distractor-based, models of the AB. It is unlikely that distractor stimuli trigger the AB or make targets more vulnerable to a loss of cognitive control if a target can be spared from the deficit despite two distractors having intervened between T1 and T2 (lag 3/30 msec). Although we suggest that target-based models are best positioned to explain the current results, the Boost and Bounce model does possess a means of accounting for this data (see [19] Figure 6). In that model, the boost is time-based so that representations of targets appearing within the boost will be enhanced. According to this account, the inhibitory bounce triggered by the two intervening distractors at lag 3/30 msec might be insufficient to overcome the temporally-defined bounce, resulting in high accuracy for T2 at lag 3/30 msec. The strict form of the distractor-based TLC model, however, cannot account for the current findings.

It is important to qualify that temporal information can only help to determine *relative* target detection accuracy at a given time, and not absolute accuracy. As is apparent in Experiment 1, targets presented at the same target onset asynchrony (180 msec) but at different presentation rates (lag6/30 msec, lag2/90 msec) will generate differing absolute accuracy levels. The value of temporal information therefore lies in its ability to indicate whether or not a stimulus will fall inside the blink period and have reduced accuracy compared with stimuli presented outside the blink at the same presentation rate.

Interestingly, we did not find clear evidence for a ‘crossover’ effect at very short SOAs. The crossover effect refers to superior T2 accuracy at SOAs less than 100 msec, but superior T1 accuracy when the SOA exceeds 100 msec [44], [45], [47], [48], [49]. However, the present study can be distinguished from those evidencing the crossover effect because targets and distractors were more easily differentiated in the crossover experiments. For example, Potter et al. [45] presented target words amongst ampersand and percentage symbols and Bachmann and Hommuk [44] presented target letters amongst a single repeated “I” distractor. The target detection task was more difficult in the current study because the variable digit distractors used here would have increased processing load, ensuring that targets could not be detected from perceptual features alone. The crossover effect may therefore be more likely to emerge when target detection does not require variable individuation of the distractor stimuli.

### Cueing in the AB

As argued above, time plays an acute role in the AB deficit. However, time is not the only important factor determining whether an AB will occur. If a target falls within the blink period but is pre-cued by another target, it will escape the detrimental effects of the AB. This study revealed that the cue-target SOA can be as brief as 30 msec or as long as 120 msec. Another target can be used as the cue, but participants need not be aware of this target’s status as a cue, nor are they required to detect this target for effective cueing to occur. And even though cueing was never detrimental to performance, cueing was most effective when the cue remained undetected, hence suggesting that future investigations make use of a cue stimulus that does not require report. Recent evidence from Harris, Benito and Dux [50] provides support for this argument. Harris et al. [50] found successful priming (which may be viewed as a form of cueing) from distractor stimuli that did not require detection. Further, distractor priming was more effective for distractors located inside versus outside the AB period, which is largely consistent with the current findings. The fact that the cue did not require detection is also consistent with motor priming work, which suggests that actions can be influenced by visual primes in the absence of conscious awareness of those primes [41], [42], [43], [51].

It is important to consider the operating mechanisms that underpin the obtained cueing effects. First, Tcue may exert a direct facilitatory effect onto the following target by initiating category-specific resources that activate the categorical ‘target’ representation. It is also possible that Tcue remains preconscious, but, as a target stimulus, Tcue initiates non-specific resources that help to optimise focal attention. For example, in Bachmann’s [52], [53] Perceptual Retouch theory, the non-specific processing of a stimulus is shown to enhance conscious perception of a following stimulus. These possibilities are not mutually exclusive and both might operate to some degree to explain the current findings. Importantly, although visual masking effects would have differed across presentation speeds, differential low level sensory masking is not particularly problematic here; sensory effects would be minimal due to the relatively large size of the RSVP stimuli (approximately 3–4°), and the figurative differences between successive stimuli. Regardless of the exact mechanisms underlying the obtained cueing effects, what is clear is that target detection in rapidly presented visual streams can benefit from immediately preceding target stimuli.

In Experiment 2, cueing was not successful for the most extreme time points employed: 30 msec/lag3 and 120 msec/lag8. At first glance these results appear consistent with Nieuwenstein’s [6] suggestion that cueing is not facilitatory if the to-be-cued target falls outside the blink period. In a number of experimental paradigms, Nieuwenstein and colleagues have demonstrated that cueing was unsuccessful – or even detrimental – when the to-be-cued target appeared more than 600 msec after T1 [6], [17]. However, the data in Experiment 2 of our current study indicate that cueing was effective for Tfinal on 90 msec/lag 8 trials (which equates to 720 msec after T1, and is therefore outside the blink period). Whether or not a late Tfinal benefits from a preceding cue likely depends upon the parameters of the task, and the level of uncued Tfinal accuracy (for example, is accuracy for the uncued Tfinal at ceiling or not). Although a systematic investigation into T2 cueing outside the blink period would be required to fully disambiguate these apparently contradictory outcomes, the current study contributes to our understanding of cueing by demonstrating that cueing *can* be effective for a target appearing outside the blink period.

Overall, the cueing findings are consistent with both target-based and distractor-based models of the AB. According to target-based explanations such as eSTST, the target cue generates a transient attentional response that acts to enhance the representation and subsequent consolidation of targets appearing within the transient window (see [6] for further discussions of cueing mechanisms within the AB). According to distractor-based models such as TLC, the cue resets the stimulus filter to process targets, hence enabling detection of the final target.

Extending an understanding of cueing within the AB, we examined the successive target advantage as a specific form of target-target cueing. Consistent with target and distractor based models, successive targets enhanced performance using 90 msec SOA. Interestingly, this effect held regardless of whether accuracy was conditionalised on T1 detection (see [40]). However, an AB was not observed using the 30 msec SOA because, at 30 msec, Tfinal accuracy across TTT and TdT conditions was equivalent. This result conflicts with distractor-based models such as TLC, which suggest that the presence of an intervening distractor on non-successive target trials should have a detrimental effect on performance. According to target-based models, all three targets would have fallen within the window of attention initiated by T1 at 30 msec presentation rates.

### Relationship to Resource-Sharing

We recently presented a correlational brain-based demonstration of resource sharing in the AB [24]. Specifically, the T1-P3b event-related potential was reduced on T2-detected trials and enhanced on T2-undetected trials. The opposite relationship was true of the T2-P3b, where amplitude was enhanced on T2-detected trials. Although a crossover effect between the amplitudes of the T1-P3b and the T2-P3b is highly suggestive of resource sharing [23], it is important to note that correlational, neural data is not capable of confirming that T1 processing directly *caused* a deficit in T2 performance.

Behaviourally, if the AB is governed by resource limitations, then an increase in Tfinal accuracy should be accompanied by a decrease in T1 accuracy [20], [22]. This appeared to be the case in Experiments 1 and 2. Resource sharing was also implicated in the comparison of Tfinal accuracy across cue-detected and cue-undetected trials in Experiment 2 (see Figure 5B). Cueing was more effective when the cue itself was not detected, suggesting that the resources required to detect the cue had to be balanced against the resources required to detect Tfinal. Importantly, the advantage of cue-undetected over cue-detected trials did not significantly interact with SOA, suggesting that resource sharing mechanisms were implicated across various speeds of presentation. If the cue-undetected > cue-detected relationship was only evident for the most rapid SOAs, basic sensory interference (for example, visual masking) might be best able to explain the benefit of cue-undetected trials. Given the current findings, we suggest that capacity limitations are implicated in the AB. Our data also resonate with recent theoretical and empirical evidence presented by Dell’Acqua and colleagues. Across three experiments, Dell’Acqua et al. [40] presented three successive targets and calculated T3 accuracy using both conditional and unconditional measures. A significant reduction in T3 performance was observed when T3 accuracy was conditional on detection of both T1 and T2, implicating resource sharing in the AB. The importance of encoding capacity limitations was further supported using a combination of simulated and empirical data [54].

Notably, the addition of a cueing target did not statistically hinder Tfinal accuracy compared with uncued Tfinal performance, indicating that the consumption of resources (in this case by the cueing target) does not always come at a cost to Tfinal. That is, the mere presence of an additional cueing target did not reduce Tfinal accuracy. Rather, the process of consolidating Tcue appeared to divert capacity-limited resources. The present study therefore reveals the importance of conditional accuracy measures in the AB paradigm, particularly with regards to cueing [28], [40]. Although resource sharing clearly plays a role in the AB, resource depletion is not the primary cause of this deficit because the AB can be overcome by placing higher resource demands on the participant. For example, by requiring them to detect three targets instead of two or by asking them to concurrently complete a second task [55]. In our opinion, resource sharing is not inconsistent with target-based explanations of the AB (but see [16] for an alternative view). It is feasible that the AB is *initiated* by target processing mechanisms but is also influenced by the degree of resources allocated to those mechanisms. Indeed, the notion that the AB is immune to resource depletion effects is problematic because it undermines the fact that the human cognitive system is inherently limited in capacity.

### Target Report Order-errors

Target report order-errors constituted an additional dependent variable in this study. Support for our order-error metric was obtained through the fact that the obtained findings were similar regardless of whether we analysed data from two-target trials, three-target trials or data collapsed over two and three target trials. In the present study, order-errors appeared to decrease as the duration between targets increased. This finding is contrasted with the accuracy data, where target detection performance decreased into the blink period, but then recovered. These results also suggest that the AB is a deficit tuned to target identity rather than target order.

The order-error findings are theoretically sensible because Wyble et al. [18] suggest that order-errors occur when numerous targets enter the encoding stage at the same time. When targets enter simultaneously, the working memory system encodes the targets’ identities but cannot preserve their episodic distinctiveness. Targets are therefore more likely to be encoded in the same episode if they appear closer together in time. Although order-errors declined with increasing target onset asynchrony, for a given SOA, errors were constant across lags if both lags occurred before the onset of the AB (Experiment 1, lags 1 and 2 at 30 msec) or if both lags occurred within the blink period (Experiment 2, lags 3 and 8 at 60 msec). A constant level of order-errors during the blink period is also sensible if we consider these errors to reflect simultaneous entry into working memory. The data suggest that a Tfinal appearing at 180 msec or 480 msec after T1 will encounter a similar level of resistance to a WM store, resulting in the same degree of order-errors. Further work is required to fully understand the nature of target report order-errors in the AB.

### Conclusions

The present study confirms and extends a number of important mechanisms governing the AB deficit. First, we confirmed the temporal nature of the AB and showed that sparing can be protracted to lag 3, provided that presentation speed is fast enough. This study also documented that the AB deficit can be overcome by a target cue that the participant need not have accurately detected. In fact, cueing was most effective when the cue was not detected. Our study also implicates resource sharing within the AB, but suggests that resource depletion does not cause the AB. We argue that these findings are most consistent with a combination of resource sharing and target-based explanations of the blink, which collectively value the contributions of optimizing transient attention in time and capacity limitations in this attentional deficit.
